# Supplementary figures and images for: Effect of Combined Methamphetamine and Oxycodone Use on the Synaptic Proteome in an In Vitro Model of Polysubstance Use
Source: Genes (Basel). 2022 Oct 8;13(10):1816. doi: 10.3390/genes13101816 (PMC9601452; doi:10.3390/genes13101816)

## Slide 1
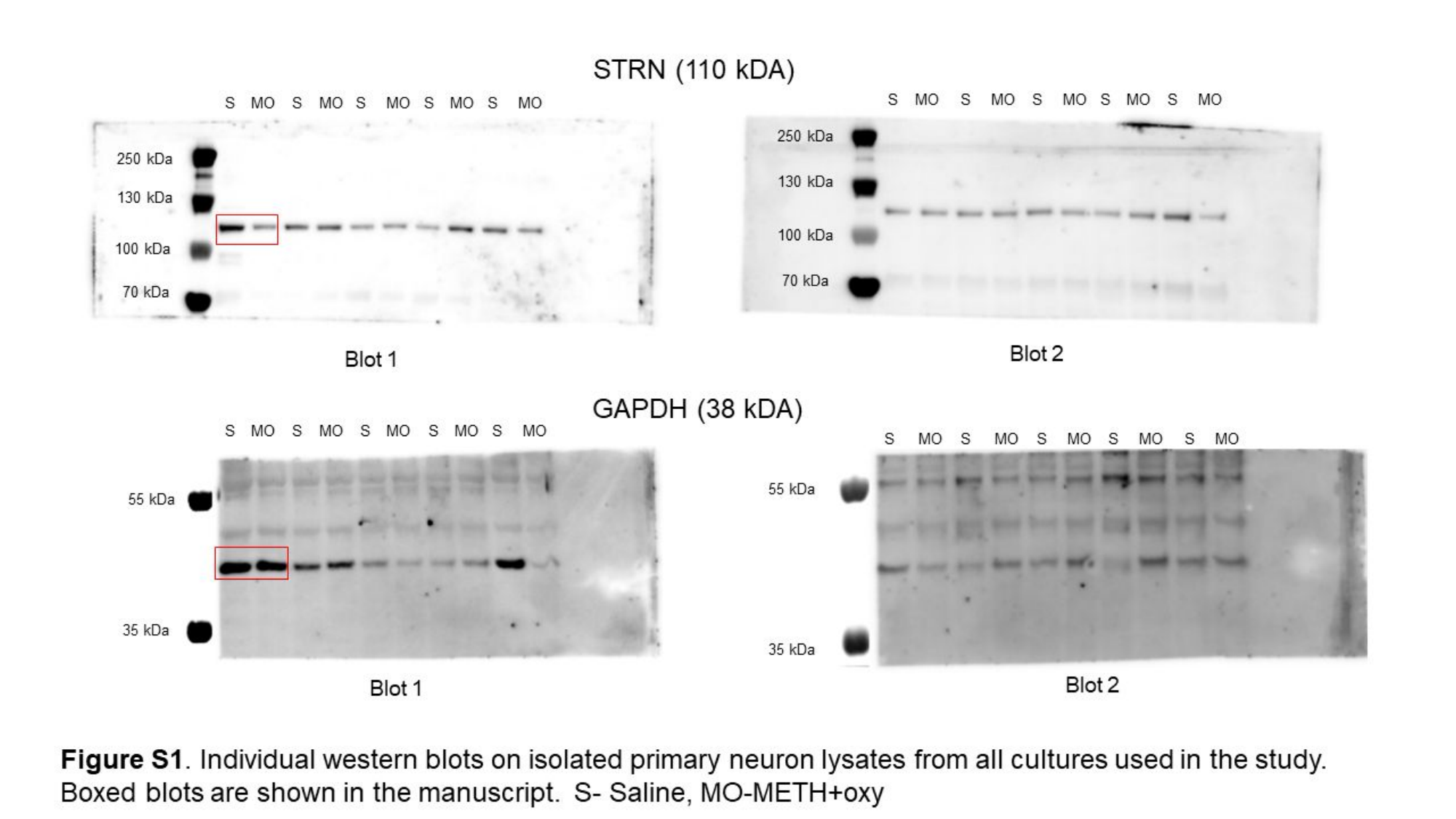

Supplement: Supplementary file 1 [file genes-13-01816-s001.zip › Figure S1.pptx]
